# The Association Between Self-Reported and Objectively Measured Energy Level in Older Adults

**DOI:** 10.1093/geroni/igaa057.579

**Published:** 2020-12-16

**Authors:** Qu Tian, Nancy Glynn, Rebecca Ehrenkranz, Briana Sprague, Andrea Rosso, Caterina Rosano

**Affiliations:** 1 National Institute on Aging, Bethesda, Maryland, United States; 2 University of Pittsburgh, Pittsburgh, Pennsylvania, United States; 3 School of Public Health, University of Pittsburgh, Pittsburgh, Pennsylvania, United States

## Abstract

Energy is an important concept in human health and diseases. Self-reported energy has been described as “the individual’s potential to perform physical and mental activity” and “the individual’s energy availability”. However, little empirical data exists on whether self-reported energy level is related to objectively measured energy level. Prior research suggests that more energy availability is associated with higher physical activity level. It remains unclear whether self-reported energy availability would be associated with objectively measured energy level, such as active energy expenditure and total energy expenditure. Using data from the Health, Aging and Body Composition Study, we identified 94 participants (mean age=86.2±2.4 y/o, 46%blacks, 52%women) with concurrent data on self-reported energy (scale 0-10) and objective energy level by the SenseWear Armband. We examined cross-sectional associations of self-reported energy with objectively measured energy and physical activity levels using Spearman correlation. Greater self-reported energy level was associated with higher daily active energy expenditure in kcal (r=0.30,p=0.004), higher METs (r=0.33,p<0.001), more minutes of physical activity (r=0.35,p<0.001), and more step counts (r=0.36,p<0.001). Self-reported energy was not associated with total energy expenditure (p=0.87) or estimated resting metabolic rate (p=0.53). Self-reported energy may reflect an individual’s activity energy expenditure but not total energy expenditure. It further supports the hypothesis that energy availability even by self-report connects to physical activity behavior. Whether self-reported energy correlates with other health outcomes warrants further investigation.

